# Prevalence of Aflatoxin- and Fumonisin-Producing Fungi Associated with Cereal Crops Grown in Zimbabwe and Their Associated Risks in a Climate Change Scenario

**DOI:** 10.3390/foods10020287

**Published:** 2021-01-31

**Authors:** Juliet Akello, Alejandro Ortega-Beltran, Bwalya Katati, Joseph Atehnkeng, Joao Augusto, Chama M. Mwila, George Mahuku, David Chikoye, Ranajit Bandyopadhyay

**Affiliations:** 1 International Institute of Tropical Agriculture (IITA), Plot 1458B, Ngwerere Road, Chelston, Lusaka P.O. Box. 310142, Zambia; j.akello@cgiar.org (J.A.); chamamebbylin@gmail.com (C.M.M.); d.chikoye@cgiar.org (D.C.); 2 IITA Nigeria, Oyo Road, Ibadan P.M.B. 5320, Nigeria; a.beltran@cgiar.org; 3 National Institute for Scientific and Industrial Research, KK Airport Road, Lusaka P.O. Box. 310158, Zambia; bkatati@nisir.org.zm; 4 IITA Malawi, Chitedze Research Station, Lilongwe P.O. Box. 30258, Malawi; j.atehnkeng@cgiar.org; 5 IITA Mozambique, Av. FPLM, Nampula P.O. Box. 709, Mozambique; j.augusto@cgiar.org; 6 IITA Tanzania, Dar es Salaam P.O. Box. 34441, Tanzania; g.mahuku@cgiar.org

**Keywords:** food safety, cereals, mycotoxins, microbial contaminants, risk assessment

## Abstract

In most sub-Saharan African countries, staple cereal grains harbor many fungi and some produce mycotoxins that negatively impact health and trade. Maize and three small grain cereals (sorghum, pearl millet, and finger millet) produced by smallholder farmers in Zimbabwe during 2016 and 2017 were examined for fungal community structure, and total aflatoxin (AF) and fumonisin (FM) content. A total of 800 maize and 180 small grain samples were collected at harvest and during storage from four agroecological zones. *Fusarium* spp. dominated the fungi associated with maize. Across crops, *Aspergillus* *flavus* constituted the main *Aspergillus* spp. Small grain cereals were less susceptible to both AF and FM. AF (52%) and FM (89%) prevalence was higher in maize than in small grains (13–25% for AF and 0–32% for FM). Less than 2% of small grain samples exceeded the EU regulatory limit for AF (4 µg/kg), while <10% exceeded the EU regulatory limit for FM (1000 µg/kg). For maize, 28% and 54% of samples exceeded AF and FM Codex guidance limits, respectively. Higher AF contamination occurred in the drier and hotter areas while more FM occurred in the wetter year. AF exposure risk assessment revealed that small grain consumption posed low health risks (≤0.02 liver cancer cases/100,000 persons/year) while maize consumption potentially caused higher liver cancer rates of up to 9.2 cases/100,000 persons/year depending on the locality. Additionally, FM hazard quotients from maize consumption among children and adults were high in both years, but more so in a wet year than a dry year. Adoption of AF and FM management practices throughout the maize value chain coupled with policies supporting dietary diversification are needed to protect maize consumers in Zimbabwe from AF- and FM-associated health effects. The higher risk of health burden from diseases associated with elevated concentration of mycotoxins in preferred maize during climate change events can be relieved by increased consumption of small grains.

## 1. Introduction

In sub-Saharan Africa (SSA), maize (*Zea mays*) and small grains, particularly finger millet (*Eleusine coracana*), pearl millet (*Pennisetum glaucum*), and sorghum (*Sorghum bicolor*), are important food security crops for millions of smallholder farmers [1,2,3]. In Zimbabwe, maize is the most important cereal crop and is cultivated throughout the country. Often, small grains are popular in the semiarid and drought-prone areas, including those of Zimbabwe, due to their outstanding hardy attributes, ability to yield better under relatively drier conditions, and superior nutritional composition [4].

Cereal crops, however, are prone to significant pre- and post-harvest losses caused by insect pests, plant pathogens, and microbial contaminants [4,5]. In many SSA regions, weather conditions, farming practices, and storage conditions favor rapid proliferation of pests and pathogens that cause significant grain losses [4,5]. Losses may include lower nutritional value, unpleasant moldy odor, discolored, rotten, shriveled grains, and mycotoxin-contaminated grains that can render the produce poisonous. All these constraints put consumers at significant health risks and preclude the crops from entering high value markets. These losses are becoming more severe across southern Africa, including Zimbabwe, where climate change-related hotter, drier conditions and erratic rainfall patterns are being experienced [6,7]. The impact of climate change on the biology of toxigenic fungi and predisposition of plants to increased mycotoxin contamination leading to elevated mycotoxin levels in crops are well documented [8,9].

Mycotoxin-producing species of the genus *Aspergillus* and *Fusarium* are common across SSA. Such fungi infect crops in the field and/or during storage [10,11,12]. Aflatoxins (AF) are produced mainly by *Aspergillus flavus* and *A*. *parasiticus*, while fumonisins (FM) are produced mainly by *Fusarium verticillioides* and *F. proliferatum*. AF and FM are among the most important mycotoxins contaminating cereal crops. Infection and toxin production are influenced by crop susceptibility, fungal species and densities, environmental conditions, farming methods, pest damage, storage practices, among others [13,14,15]. Consumption of contaminated produce is hazardous with acute and/or chronic exposure potentially leading to liver cancer, esophageal cancer, neural tube defects, stunting, immunosuppression, infertility, among other maladies [16,17,18,19,20]. Due to high dietary exposure [21,22,23,24,25], harmful and sometimes lethal effects [26,27], regulatory limits have been set for both groups of toxins [28,29] to reduce exposure risks and protect human health [30].

Within SSA, studies describing multiple occurrences of mycotoxin producers and associated toxins in marketed foods and feeds have focused mostly on maize- and groundnut-based products [10,11,31,32,33,34]. Other crops such as finger millet, pearl millet, and sorghum have received relatively little attention. In Zimbabwe, the focus has been maize and groundnut as well [10,17,35,36,37,38,39,40]. Information on the safety of other staple and food security crops, particularly sorghum and the millets grown in Zimbabwe is limited.

Periodic surveillance coupled with awareness creation and advocacy for safer foods remains a key measure for facilitating behavioral, attitude, and mindset changes for reduced mycotoxin contamination and subsequent exposure [41]. Mycotoxin prevalence data also provides guidance to the decision-making process of setting and revising regulatory limits. With a potential heightened impact of climate change on mycotoxin levels [14,42], there is a need to assess the current status of mycotoxins in small grains. The present study, therefore, aimed at assessing frequencies of mycotoxin-producing fungi in maize, sorghum, pearl millet, and finger millet cropped and traded in Zimbabwe during 2016 and 2017, and to quantify levels of AF and FM in those crops. In addition, the study estimated the exposure risks from AF and FM contamination, and assessed mycotoxin impact on trade. Information from this study will be useful to update knowledge of mycotoxin risk of various cereals and inform policies on diet diversification, especially as a food safety coping strategy in response to climate change, in Zimbabwe and other countries where the four crops are consumed.

## 2. Materials and Methods

### 2.1. Survey Sites

**Figure 1:**
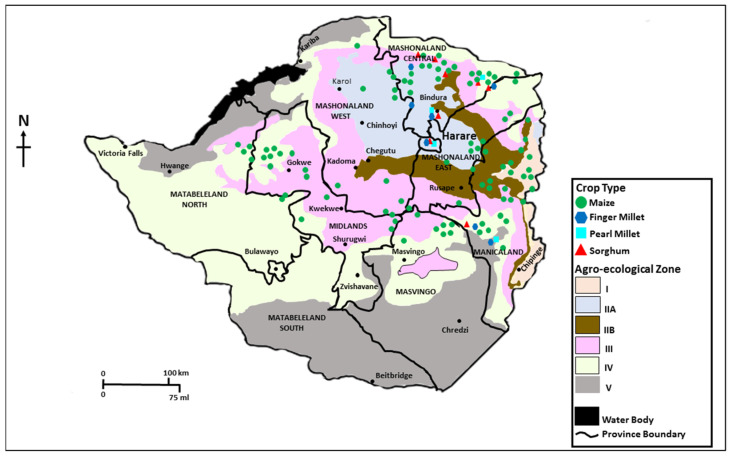
Map of Zimbabwe showing agroecological zones and locations from where maize, finger millet, pearl millet, and sorghum grain samples were collected during the 2015/2016 and 2016/2017 seasons.

Samples of maize and small grains were collected across four agroecological zones (AEZs; i.e., regions IIA, IIB, III, and IV) of Zimbabwe (Figure 1). Regions IIA and IIB receive 750–1000 mm of rainfall per year, while regions III and IV receive 650–800 mm and 450–650 mm, respectively. Three provinces were sampled: Manicaland, Mashonaland Central, and Midlands. In Manicaland, samples were collected from five districts, Buhera, Mutare, Makoni, Mutasa, and Nyanga. In Mashonaland Central, samples were collected from Rushinga, Muzarabani, Guruve, and Mt. Darwin districts. In Midlands, crops were sampled from Chirumanzi, Gokwe South, and Kwekwe districts (Figure 1). Except for Midlands in which sampled districts cut across AEZs III and IV, the rest of the sampled provinces spanned across the four AEZs. Samples were collected at harvest in June 2016 and May 2017 and during storage in October 2016 and September 2017. A prolonged dry spell and drought occurred during the 2015/2016 cropping season (avg. precipitation = 367 mm; range = 147–620 mm) compared to the 2016/2017 season (avg. = 536 mm; range = 260–656 mm).

### 2.2. Samples

A total of 800 maize samples and 180 small grain samples (60 each of sorghum, finger millet, and pearl millet) were collected during the study period to determine both the prevalence of AF- and FM-producing fungi and levels of the two groups of toxins. In each cropping season, 200 maize samples were collected at harvest while the crop was still in the field or from drying sheds, and another 200 samples of shelled maize were collected during storage (4–5 months after harvest). At harvest, 30 maize cobs were randomly picked from the field or drying shed, shelled by hand and mixed to form a 5 kg composite sample. For stored maize, a 5 kg composite sample was sourced randomly from three to five bags kept within the homesteads of farmers. Small grain samples of 2 kg each were collected from farmers’ stores, local markets, or grain marketing boards. All samples were geo-referenced and transported to the laboratory of the Department of Research and Specialist Services in Harare, Zimbabwe.

### 2.3. Sample Processing

Maize samples collected at harvest were dried in a hot air oven at 45 °C for 48 h. For each maize sample, a 2.5 kg subsample was obtained and shipped along with small grain samples to the IITA Pathology laboratory in Lusaka, Zambia, for further processing and analysis. Samples were stored at −20 °C until analysis. For microbiological analyses using whole grains, 200 g of grain was subsampled from each sample. The remaining sample (≈2.3 kg for maize and ≈1.8 kg for small grains) was milled using a coffee mill grinder (Bunn-o-Matic Corporation; Springfield, IL, USA), mixed thoroughly, and a 250 g subsample obtained for mycotoxin and microbiological analyses for *Aspergillus* characterization (see below). The grinder was thoroughly washed with 70% ethanol between samples to prevent cross contamination of both toxins and microorganisms.

### 2.4. Fungal Characterization

Initially, fungi from the grains were characterized on low strength potato dextrose agar (PDA, HiMedia Laboratories GmbH, Einhausen, Germany; 10 g/L) amended with 5 mL/L chloramphenicol (10 mg/L). After autoclaving (121 °C, 20 min) and cooling, 10 mg/L dichloran and 50 mg/L streptomycin sulphate were added before dispensing into Petri plates. For each 200 g whole grain subsample, 50 g of maize or 5 g of small grains were washed in 1% sodium hypochlorite—for surface-disinfection purposes—for 5 min and rinsed three times with sterile distilled water. Six grains of maize and 10 of the other cereals were placed onto the medium (20 plates per sample). Plates were incubated at 25 °C (7 d, dark), and then fungal colonies were enumerated. Based on macroscopic and microscopic characteristics, the fungi were classified as *Fusarium* [43], *Aspergillus* [44], *Penicillium* [45], or other genera.

Further classification of *Aspergillus* fungi to the species level was conducted on modified Rose Bengal agar (MRBA; 2% Bacto agar, 3 g sucrose, 3 g NaNO_3_, 0.75 g KH_2_PO_4_, 0.25 g K_2_HPO_4_, 0.5 g MgSO_4_ 7H_2_O, 0.5 g KCl, 1 mL micronutrients, 5 mL Rose Bengal, pH 6.0) amended with dichloran and streptomycin sulphate [44]. In total, 1–5 g of milled sample was suspended in 10 mL sterile distilled water and known volumes of the suspension plated. Plates were incubated at 31 °C (3 d, dark). Thereafter, *Aspergillus* colonies were enumerated, and 15 colonies transferred to 5-2 agar media (5% V8 Juice (Campbell Soup Company, Camden, NJ, USA); 2% agar, pH 6.0). Plates were incubated at 31 °C (5 d, dark). *Aspergillus* fungi were assigned to their corresponding species based on growth on specialized media [44], morphological characteristics, and spore ornamentation [46]. For microscopic assessment, wet mounts were prepared, and the slides examined using a light microscope at a magnification of 100×. Isolates with abundant small (<400 µm) sclerotia and scarce olive green conidiation were classified as S morphotype, while isolates with copious olive green conidiation and few, large sclerotia (>400 µm) were classified as the *A. flavus* L morphotype. S morphotype could be any of the following species: *A. flavus*, *A. aflatoxiformans*, *A. minisclerotigenes*, or an unknown species. Isolates with dark green, rough spores were classified as *A. parasiticus*. Isolates producing abundant tan conidia were classified as *A*. *ochraceous* and isolates producing abundant black conidia were classified as *A*. *niger*.

### 2.5. Mycotoxin Quantification

In each of the 180 small grain and 800 maize samples, AF and FM were extracted using procedures outlined in the Neogen protocol handbook (Reveal Q+ for aflatoxin and Reveal Q+ for fumonisin, Neogen Corporation, Lansing, MI, USA). Briefly, for each sample, 50 g of milled sample was weighed and transferred to a 500 mL Pyrex bottle. Then, 250 mL of 65% ethanol was added to the bottle, capped, and the content mixed by swirling. Bottles were placed on a MaxQ 2000 orbital shaker (Thermo Fisher Scientific Inc., Bartlesville, OK, USA), and shaken at 500 rpm for 3 min. Extracts were filtered through a Whatman No. 1 filter paper (Whatman International Ltd., Maidstone, UK). The toxins were quantified using the Neogen AccuScan Lateral Flow Device. The AccuScan total AF lower limit of detection (LOD) is 2 μg/kg while the upper detection limit (UDL) is 150 μg/kg. For FM, the LOD is 300 μg/kg and UDL is 6000 μg/kg. Samples exceeding UDLs were diluted with 65% ethanol and the analysis was repeated. AF- and FM-contaminated reference materials from Neogen were repeatedly analyzed after every 20 samples for quality control checks. Additionally, maize, millet, and sorghum samples were spiked with pure AF or FM and recovery rates of over 90% were obtained. To validate the AccuScan results, 7.5% of maize samples were analyzed with high-performance liquid chromatography (HPLC) in an accredited analytical laboratory, the Perishable Produce Export Certification Board (PPECB) in South Africa.

### 2.6. Risk Assessment: Dietary Exposure and Potential Health and Trade Impact

AF and FM levels quantified in all samples were used to assess dietary exposure. The average per capita consumption rates used in the analysis was 248 g per person per day (g/p/d) for maize, 8.71 g/p/d for both finger millet and pearl millet, and 18.04 g/p/d for sorghum [47]. The average body weight (bw) for adults and children were set at 60.7 and 25.0 kg, respectively [47]. Aflatoxin B1 (AFB1) data was unavailable for the simulation of liver cancer potency attributed to this toxin; therefore, a best available estimate approach, employing the common ratio and maximum permissible limit settings used worldwide of 2:1 for total aflatoxin (AF) to AFB1, was used. Often, AFB1 is estimated to be at least 50% of the quantity of AF as demonstrated in some studies [48]. Thus, for each sample, AFB1 value was obtained by halving the total AF value. Based on probable daily intake (PDI), dietary exposure to total AF and AFB1 was calculated by multiplying mean concentration in each crop by the consumption rates of each commodity and the obtained value was divided by the bw value as shown below (Equation (1)). To determine mean AF values that were used to study risk assessment, AF concentration in all samples with below the instrument LOD were replaced with instrument LOD value of 2 µg/kg. The margin of exposure (MOE) was computed using a benchmark dose lower limit (BMDL) of 170 ng/kg bw/day [49] for AF (Equation (2)).

PDI (μg/kg bw/day) = (*μ* × C)/bw (1)

where *μ* = mean AF or AFB1 or FM concentration; C = quantity of maize or small grains consumed in Zimbabwe.

MOE = BMDL/PDI(2)

To estimate the potential impact of maize AFB1 and FM exposure on health and trade, storage datasets for AF (*n* = 400) and both harvest and storage datasets for FM (*n* = 800) were used. Estimation of the posterior risk exposure for AFB1 was carried out using a Bayesian model [50,51], based on an acute exposure estimation on hepatocellular carcinoma (HCC) effect of the toxin on humans [52]. AF-induced HCC rates (defined as Liver Cancer (LC) rates in Equation (3) below) were computed by multiplying AF potency as shown in Equation (3) by exposure risk (PDI) described in Equation (1). The average potency (AP) was derived from Equation (4) on the basis of prior values of proportions of hepatitis B virus (HBV) prevalence rates for individuals positive for the HBV surface antigen (HBsAg; a biomarker of chronic HBV infection) and HBsAg-negative individuals [27]. The proportions are estimated at 0.144 and 0.856, respectively, for Zimbabwe [52]. Therefore, in the model, AFB1 carcinogen potency prior value of 0.05176 cases per adult population in ng/kg bw/day for Zimbabwe was used based on the national HBV prevalence of 14.4%, and a national population of 13,076,978 [52]. Briefly, studies have shown that the risk of LC in individuals exposed to HBV infection and aflatoxin is 30 times greater as compared to individuals exposed to aflatoxin only [53] due to a synergistic effect of the two factors.

LC = PDI × AP(3)

where AP = estimated national carcinogen potency to AFB1 exposure in ng/kg bw/day (based on cases/100,000 persons/yr (CPY)).

AP = (0.3 × proportion of HBsAg-positive prevalence rate) + (0.01 × proportion of HBsAg-negative prevalence)(4)

The cut-off points for simulated AFB1 to assess the impacts for maximum limits (MLs) were set at 0 (no regulation), 4 μg/kg (EU) [54], a provisional value of 10 μg/kg (simulation using the cutoff value for AF in Zimbabwe) [28], all of which would translate into 0, 8, and 20 μg/kg for AF, respectively. ALARA (as low as reasonably achievable) values [55,56] were also set for each province. ALARA value was derived as the mean of samples up to the 95th percentile in the range of samples from lowest to highest AF concentration. For FM, the MLs cutoff points were set at 0 (no regulation), 2000 μg/kg (Codex) [28], and ALARA values of 885.6 and 2873 μg/kg for 2015/2016 and 2016/2017 seasons, respectively. Risk exposure to FM was estimated on the basis of a hazard quotient (HQ), defined as the ratio of estimated exposure to FM (Equation (1)) divided by the Provisional Maximum Tolerable Daily Intake (PMTDI) for FM that was set at 2 μg/kg bw/d [57]. Similarly, as with AF, the LOD value was used to determine mean FM concentration such that a sample whose value was below the instrument LOD of 300 µg/kg was scored as equivalent to 300 µg/kg.

### 2.7. Data Analysis

Incidences of *Fusarium*, *Aspergillus*, *Penicillium*, or other fungal species on grain were calculated as the number of kernels with visible growth of the corresponding genera divided by total number of plated kernels × 100. For mycotoxin data, all samples with AF and FM below 2 and 300 μg/kg, respectively, were given a value of zero. Thereafter, AF and FM data were subjected to a mixed model analysis of variance with location (districts) considered as a random effect. Since there were neither seasonal nor sampling time effects on AF levels, the AF data sets for the two seasons were pooled and used in descriptive analysis to determine the mean values ± SD. Data for FM were not pooled. To determine the impact of mycotoxins on health, the data was computed as described under Equation (1) for FM and Equations (1)–(4) for AF. Unlike for the mixed model analysis, datasets used for computing exposure risks were modified by replacing data points whose values were below limit of detection by 2 µg/kg (AF) and 300 µg/kg (FM). All statistical analyses were conducted using SAS statistical software version 9.4 (SAS Institute, Cary, NC, USA).

## 3. Results

### 3.1. Fungal Characterization

Incidences of fungi on grains were relatively moderate, with 27–60% of samples not yielding any fungus. Fungal incidence was highest in maize, followed by finger millet, sorghum, and lowest in pearl millet (Table 1). The predominant genera encountered were *Fusarium*, *Aspergillus*, *Penicillium*, *Paecilomyces*, *Trichoderma*, *Phoma*, *Cercospora*, *Rhizopus*, and yeasts (Table 1). Distributions of fungal species varied across crops. In small grains, over 70% of the fungi belonged to species of *Phoma* and *Cercospora*, while for maize 84% belonged to *Fusarium*, *Aspergillus*, or *Penicillium* spp.

**Figure 2:**
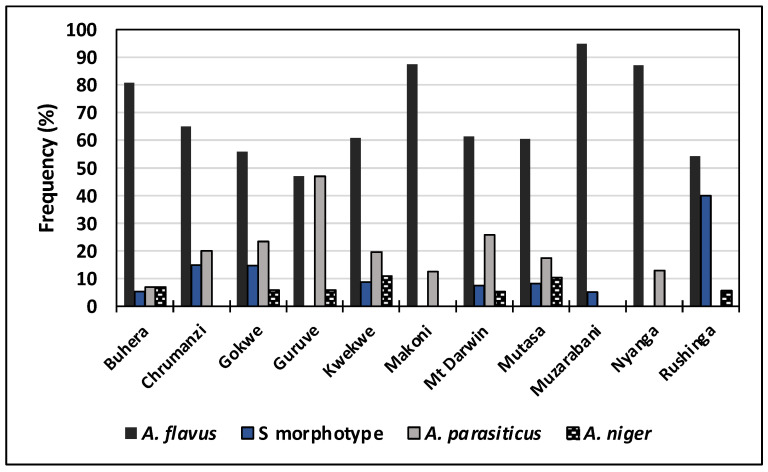
*Aspergillus* species distribution in 11 districts of Zimbabwe. Values are for mean incidence detected in maize, sorghum, pearl millet, and finger millet grain samples (*n* = 740 isolates) collected during two seasons.

Subsequent *Aspergillus* characterization revealed several species associated with maize and small grains: *A*. *flavus*, *A*. *parasiticus*, *A*. *ochraceous*, *A*. *niger*, and S morphotype fungi. In general, maize contained higher densities of *Aspergillus* spp. than small grains (data not shown). Irrespective of the crop, *A. flavus* L morphotype occurred at higher frequencies (>67%) than *A*. *parasiticus* or the S morphotype fungi, which were isolated in relatively equal proportions across crops (Table 2). The least frequently isolated *Aspergillus* species were *A. niger* (occurring in all crops) and *A*. *ochraceous* (occurring only in maize). The L morphotype, found in all districts, was the predominant species in all locations. On the other hand, *A*. *parasiticus* was isolated in all districts except in Muzarabani and Rushinga. The S morphotype fungi were found in eight of the 12 districts. In Guruve, L morphotype and *A*. *parasiticus* occurred in equal proportions, while in Rushinga the proportions of the L morphotype exceeded that of the S morphotype fungi by about 10% (Figure 2).

### 3.2. Mycotoxin Levels in Grains

Overall, the incidence of AF contamination was relatively low in small grains (13% to 25%), but moderate (≈50%) for maize (Table 3). Maximum AF levels were 1369 μg/kg for maize, 58.0 μg/kg for pearl millet, 4.3 μg/kg for sorghum, and 3.6 μg/kg for finger millet. The average AF levels were 16.8 μg/kg for maize, 2.5 μg/kg for pearl millet, and <2 μg/kg for both finger millet and sorghum. Over 80% of the maize had AF levels within the EU (4 μg/kg) and 89% within Zimbabwe (10 μg/kg) regulatory limits (Table 3). On the other hand, all small grain samples except for one and two pearl millet and sorghum sample(s), respectively, contained safe AF levels.

Analysis of maize AF data both at harvest and after storage across the two seasons revealed no effect of season (*P* = 0.2895), sampling time (*P* = 0.7461), nor AEZ (*P* = 0.7869). Equally, no interaction occurred between these variables (*P* ≥ 0.6631 ≤ 0.9377). Though nonsignificant, maize from Muzarabani had the highest mean AF level. Nyanga district, with less than 60% AF positive maize samples, showed the lowest mean AF level. Rushinga, Buhera, and Muzarabani, had 12–28% of maize samples with AF levels above the US regulatory limit (20 μg/kg; Table 4).

FM was either undetected or detected at very low levels in small grain samples. However, most maize samples (89%) had FM. For positive samples, FM ranged from 300 to 40,000 μg/kg in maize, 300 to 1500 μg/kg in finger millet, and 300 to 2800 μg/kg in sorghum. Mean FM levels were 2383, 412, and 127 μg/kg, for maize, sorghum, and finger millet, respectively. Less than 10% of the millets and sorghum had FM above the EU regulatory limit, while 54% of the maize exceeded that limit (Table 3).

**Figure 3:**
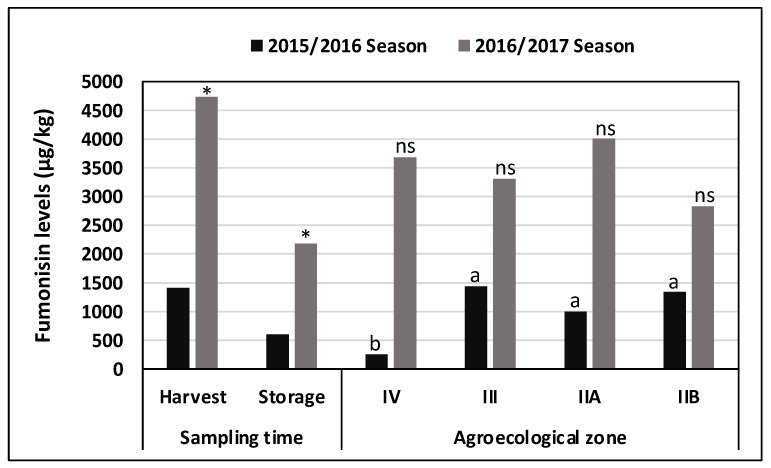
The effect of sampling time and agroecological zones (AEZ) on fumonisin (FM) contamination in maize during two cropping seasons. * indicates significant differences (*P* < 0.05, Student’s *t*-test) in FM between harvest and storage within each cropping season. FM content was compared among AEZs in each individual cropping season; bars with the same lowercase letters are not significantly different (*P* < 0.005, Tukey’s Studentized Range (HSD) Test); ns: no statistical difference. The precipitation levels in each AEZ are 450–650 mm for AEZ IV, 650–800 mm for AEZ III, and 750–1000 mm for AEZs IIA and IIB.

Season and sampling time significantly influenced maize FM content (season: F = 46.48, df = 1, *P* < 0.0001; sampling time: F = 22.12, df = 1, *P* < 0.0001). However, FM levels were not influenced by AEZ (F = 0.65, df = 3, *P* = 0.5854). Compared to the 2015/2016 season, FM level was significantly higher in the 2016/2017 season. Maize FM level, however, depended on the interaction between season and sampling time (F = 5.91, df = 1, *P* = 0.0153). In both cropping seasons, FM content in samples collected at harvest was higher than in samples collected after storage (Figure 3). Additionally, FM levels in maize were influenced by the interaction between season and AEZ (F = 3.13, df = 3, *P* = 0.0252). At harvest, maize from the 2015/2016 season contained 3–6-fold significantly lower FM levels in the hotter and drier region IV AEZ when compared to the other three AEZs. For the 2016/2017 season, there was no statistical difference in FM levels across AEZs (Figure 3). Across districts, maize samples from Makoni, Mutasa, Chirumanzi, and Kwekwe had >90% of samples containing FM levels within regulatory limits (Table 5). Muzarabani and Nyanga districts harbored the largest proportion of samples containing FM above the regulatory limit.

### 3.3. Exposure Risk Assessment: Probable Daily Intake and Mycotoxin Health and Trade Impacts

In all seasons and sampling time, dietary exposure to AF was highest among maize consumers with PDI reaching a maximum of 5593 ng/kg bw/d for total AF and 2797 ng/kg bw/d for AFB1. On the other hand, very low dietary exposure was observed among millet and sorghum consumers with PDI not exceeding 10 ng/kg bw/d (Table 6). Consequently, maize with a relatively small MOE (<5) posed higher hazardous health risks to the public when compared to the other cereal grains. Over the two cropping seasons, consumption of the sampled maize had an estimated potential LC rate of 1.8 CPY. Maximum PDI was highest in region IV AEZ and lowest in region IIA AEZ. Consumption of maize from region IV AEZ had an estimated LC rate of 4 CPY and posed the greatest dietary exposure risk to consumers (Table 7). Though exposure risks across districts followed a similar pattern to AF prevalence, the MOE values in Buhera, Chirumanzi, and Muzarabani were relatively small (1–5), highlighting a potentially higher exposure risk for consumers in these districts. Overall, maize from Muzarabani in region IV posed higher health risks to the public, potentially leading to at least 9 LC CPY (Table 8).

Across provinces, consumption of AF-contaminated maize stored for 4–6 months posed health risks with calculated LC rates of 0.4–2.1 CPY for the pooled data for 2015/2016 and 2016/2017 seasons (Table 9). The highest risk of developing LC was observed in Mashonaland followed by Manicaland (Table 9). Enforcing stringent measures/regulations of 4 μg/kg (EU) would reduce LC risks throughout the provinces to 0.2 CPY from the maximum value of 2.1 CPY (no regulation) for the most affected province. However, the potential for grain rejection in trade increased from 0% to up to 13.9% (for the most affected province). Employing the ALARA limit showed a 5-fold risk reduction for the most affected province from 2.1 CPY (no regulation) to 0.4 CPY. The potential for grain rejection in this case increased from 0% to 5.6%. Using the provisional value of 10 μg/kg as a limit, reduced the risk exposure to 0.3 CPY across the provinces. However, this increased the potential for grain rejection from 0% to 8.6% (for the most affected province). 

**Figure 4:**
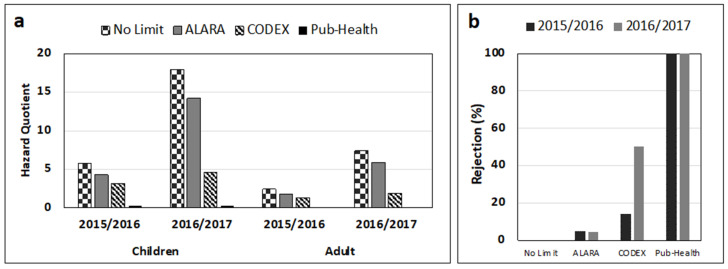
Estimated hazard quotient (**a**) and potential rejection of maize grains (**b**) due to fumonisin contamination in different provinces of Zimbabwe following the 2015/2016 and 2016/2017 cropping seasons. Codex value of 2000, 886, and 2873 μg/kg of ALARA (2015/2016 and 2016/2017 seasons, respectively), and a simulated Public Health value of 67 µg/kg for maximum limits were considered.

For FM, the estimated HQ in both seasons was 2.4–17.9 times the PMTDI value across all age groups with season 2016/2017 exhibiting higher risks. Based on mean FM levels, the mean HQ amongst children (5.9 and 17.9 for seasons 2015/2016 and 2016/2017, respectively) was higher than that for adults (2.4 and 7.4 for seasons 2015/2016 and 2016/2017, respectively) (Figure 4a). The highest FM exposure risks and proportion of noncompliant samples were recorded after 2016/2017 season in all provinces. Furthermore, this season had greater potential Codex standard guided grain rejections at 50.3% compared to 14.3% for the 2015/2016 season (Figure 4b).

## 4. Discussion

Ear and panicle rot fungi, predominantly species of *Fusarium*, *Aspergillus*, and *Penicillium* were recovered from maize, sorghum, pearl millet, and finger millet grown in Zimbabwe between 2015 and 2017 in relatively high frequencies. Studies conducted in Zimbabwe and elsewhere, show that *F. verticillioides*, *A. flavus*, and *A. parasiticus* are major mycotoxin producers contaminating cereals [32,37,58,59,60,61,62,63]. Results from our study revealed that the maize mycobiome was dominated by *Fusarium* and *Aspergillus* spp., while in small grains other filamentous fungi dominated, mainly *Phoma* and *Cercospora* spp. These findings are similar to studies in West Africa and elsewhere in which sorghum, pearl millet, and finger millet were reported as less susceptible to AF producers compared to maize [12,31,32]. Among the aflatoxin-producing fungi, the *A*. *flavus* L. morphotype was the predominant species on both maize and small grains. Studies conducted nearly a decade ago similarly reported *A*. *flavus* as the predominant *Aspergillus* spp. infecting maize from northern Zimbabwe [36]. In neighboring Zambia and Mozambique, *A*. *flavus* L morphotype was also reported as the most frequently isolated species in maize, followed by *A*. *parasiticus* [59,63].

For the first time, we report the prevalence of FM and AF in finger millet that were locally produced and traded in Zimbabwean informal markets. AF and FM co-occurred in all crops except for pearl millet, which had no FM. Detection of both AF and FM in maize at harvest indicates that contamination occurs at the preharvest stage. For small grains, it is unclear if the contamination occurred at preharvest. In the current study, FM incidence and concentration were higher than that of AF in maize and sorghum but not for the millets. High FM content compared to AF in maize was previously reported in Zimbabwe [38,39]. In the millets, AF incidence of 13–25% was recorded while FM in these crops ranged from 0% to 3.3% (Table 3). 

Though low mycotoxin contamination of small grains was recorded, we observed moderate AF (≈50%) and high FM (≈90%) incidence in maize (Table 3). AF content in maize was considerably higher than in the other crops (7–19-fold higher) and most maize had FM. Thus, consumption of maize in Zimbabwe significantly exposes people and/or livestock to both AF and FM. This has been previously documented [17]. Consumption of the less AF- and FM-susceptible small grains to diversify diets and reduce exposures to both groups of toxins should be encouraged [12,31]. Surveys conducted in various regions indicate that millet and sorghum are less prone to AF and FM than maize [12,31,40]. In West Africa, Bandyopadhyay et al. [31] found maize collected in the field to harbor 4–8 times higher AF levels than sorghum or pearl millet. Similarly, in Ethiopia, variable levels of several mycotoxins contaminating small grains were reported [32]. In Nigeria, high FM content in maize was related to higher frequencies of FM-producing fungi compared to sorghum or pearl millet [12]. Contrary to our findings, a mycotoxin study in Zimbabwe [61] found FM in 61% of sorghum samples but detected no AF. The contrast may be due to differences in seasonal mycotoxin fluctuations, sampling time, and location, which often influence mycotoxin levels.

Though AF levels were not influenced by sampling time, AEZ, or season, FM levels in maize differed across AEZ and cropping season. Generally, environmental factors (especially drought stress) dictate the prevalence and severity of mycotoxin contamination in food and feed crops [62,64]. In our study, the highest AF content was observed in the hotter and drier parts of Zimbabwe, which receive 450–600 mm rainfall annually [65]. Similarly, though not statistically significant, we observed that maize collected after the 2015/2016 cropping season and that received low rainfall (368 mm) had higher AF content than maize collected after the 2016/2017 cropping season (rainfall: 536 mm), which concurs with studies conducted elsewhere [23,64,66]. For instance, in neighboring Zambia, AF content in maize and groundnut was higher in a low rainfall and drier region compared to a cool and wet region [64]. Similarly, maize and sorghum grown in semiarid tropical areas of Kenya were more vulnerable to AF contamination than in temperate areas [67]. Likewise, Ding et al. [23] reported that groundnut cultivated in areas with low rainfall and relatively high daily mean temperature (≈25 °C), or exposed to heat stress (26–31 °C) during the last month of the growing season resulted in high AF levels [66]. Our study provides additional evidence on the influence of weather and environmental stress in favoring the proliferation of AF-producing fungi and AF severity.

Millets were remarkably less contaminated with AF and FM (Table 3). A hotter, drier season as well as a more humid season contributed to higher AF and FM, respectively, in maize and/or sorghum. Climate change is known to cause higher mycotoxin-producing fungi build up and mycotoxin contamination in susceptible crops [8,9]. Higher temperatures, greater CO_2_ concentrations, erratic rainfall patterns, all are known to increase crop susceptibility to mycotoxin contamination. However, regardless of the weather fluctuations that occurred in Zimbabwe during the study, these did not readily impact mycotoxin levels in the millets. It is likely that mycotoxin contamination of prone crops, especially maize and, to some extent sorghum, will increase in the future due to climate change. Cultivation of millets is a valuable option to palliate the effects of climate change in a sustainable manner [68]. Research to understand the potential and limits of the millets in resisting mycotoxin contamination as a result of current and future environmental fluctuations caused by climate change should be conducted.

Generally, daily exposure to mycotoxin levels exceeding 1 µg/kg bw/d (AF) and 2 mg/kg bw/d (FM) are known to have adverse effects on health [17,20,22]. Furthermore, for carcinogenic substances like AF, MOE values ≥ 10,000 are considered protective. In the present study, all the cereal grains had mycotoxin levels and MOE values less than 10,000 signifying that all posed health hazard to consumers. However, maize’s high estimated PDI (up to 2797 ng/kg bw/d) along with a relatively small MOE value (≤5) for AF, and high FM HQ (>1) suggested that maize consumers compared to consumers of small grains faced a potentially higher risk of developing illnesses related to AF (e.g., liver cancer, child stunting, immunosuppression) and FM (e.g., esophageal cancer, neural tube defects, immunosuppression). 

Data on FM exposure revealed a high risk of chronic exposure to this toxin for all age groups over the two seasons. The 2016/2017 cropping season, with relatively high rainfall and wet conditions, exhibited a higher FM average (3609 μg/kg) compared to the 2015/2016 cropping season (1191 μg/kg) and subsequently higher HQ values. For the most vulnerable group, like children, the HQ of 5.9 (2015/2016 cropping season) and 17.9 (2016/2017 cropping season) exceeded the Codex PMTDI for FM. Likewise, the HQ values of 2.4 and 7.4 in cropping seasons 2015/2016 and 2016/2017, respectively, were high to potentially expose adult consumers to FM. Similarly, a study by Hove et al. [17] indicated a high risk of exposure to FM among populations in Zimbabwe. However, Hove and coworkers reported a relatively lower exposure to FM contamination (HQ up to 2.7) compared to our findings (HQ up to 7.4). This variability could be attributed to seasonal differences as well as the locations from where the samples were obtained, all of which are factors known to influence mycotoxin build up. 

Overall, the most AF-affected region was AEZ IV followed by III. Those AEZ of Zimbabwe are relatively hotter and drier; thus, maize from those AEZ may pose AF-exposure risks to consumers in the region. According to Wild et al. [69], all SSA countries have favorable conditions supporting high proliferation of mycotoxin-producing fungi and mycotoxin production, and as such inhabitants are highly exposed to mycotoxin health risks. Depending on the crop, the AEZ, location (district), and any possible intervention (e.g., enforcement of regulations), the estimated cancer risks of 0.2–9.2 CPY were recorded over the 2-year period. We report that enforcing stringent regulations along with dietary diversification to small grains would significantly reduce exposure risks and the negative health impacts associated with the two toxins [24]. Wu et al. [30] reported that enforcement of current regulatory AF standards of 4–20 µg/kg can adequately protect human health worldwide, except in Africa, if the protection level of 1 in 10,000-lifetime HCC cases in the population are desired. Nonetheless, application of ALARA or the adopted FM Codex limit of 2000 µg/kg for both seasons may not sufficiently protect consumer groups (Figure 4a).

From a trade perspective, the observed mycotoxin levels would have resulted in significant rejection of maize grain lots. Such rejections can lead to economic losses for the nation. For instance, we observed that the current Codex ML of 2000 µg/kg for FM, despite not translating into sufficient protection for the different consumer groups, translated into potential grain rejections of 14.3% and 50.3% for the two successive cropping seasons, respectively (Figure 4b). Similarly, up to 28% maize grains did not meet the Codex standard for AF and were liable for rejection (Table 4). Thus, it is imperative that Zimbabwe establishes and enforces appropriate interventions [24] including strict regulations and policies that support dietary diversification if consumer exposure risks to mycotoxins and associated negative health and trade impacts are to be minimized. However, the practicality of enforcing regulation in SSA settings has been questioned [67]. It is necessary to make farmers aware of the dangers of mycotoxins and enable them to implement proven technologies for mycotoxin mitigation. Greater mycotoxin reductions and exposure would be achieved with policy and institutional support.

## 5. Conclusions

Our study revealed that AF- and FM-producing fungi along with their secondary metabolites, i.e., AF and FM, commonly co-occur in four cereal grains grown in Zimbabwe. AF and FM content depended on the crop, with AF levels in maize being 7-fold, 15-fold, and 19-fold higher than that detected in pearl millet, sorghum, and finger millet, respectively. This confirms a higher susceptibility of maize to these contaminants relative to small grains, while at the same time stresses the need for dietary diversification to minimize mycotoxin exposure under climate change scenario. Mycotoxin incidence and prevalence was influenced by seasonal and agroecological factors. Crops from drier and hotter localities exhibited relatively higher AF levels, while a relatively wetter season/AEZ supported higher FM levels in maize. These results point to the need for routine surveillance and monitoring of these commodities by food regulators. The MOE for all the crops were below the established protective values of 10,000, signifying that all the crops posed detrimental health hazards to consumers. However, maize with exposure estimates of 5.5–177.7 ng/kg bw/d and relatively low MOE values showed that consumers were at risk of developing AF-related illnesses with potential cancer burdens of 0.3–9.2 CPY against small grains values of less than 0.1 CPY. Enforcement of regulations would have reduced AF cancer burdens by at least 9%. For FM, the estimated HQ exceeded the Codex PMTDI of 2 µg/kg bw/d across all provinces in both seasons. It may appear that the use of 300 µg/kg LOD for FM would result in higher HQ value compared to HQ value calculated using zero, the two HQ values were very similar to each other (data not shown). The FM level of exposure and risk posed was higher in children than adults signifying the vulnerability of this age group to mycotoxin’s detrimental health effects (immunity impairment and growth retardation) attributed to chronic exposure.

Maize contamination with the two mycotoxins had potential of hindering international/regional trade by up to 13.9% for AF-related rejections and 100% for FM-related rejections. Thus, to reduce potential health hazards and boost regional or international trade, inclusion of less susceptible food crops like sorghum and millets in the diets should be promoted along with other mitigation measures like enforcement of regulations, good agricultural and storage practices, as well as advocacy and awareness campaigns. In general, millets (and to some extent sorghum) appeared to be less affected by environmental fluctuations and accumulated less mycotoxins. This may be important to consider for the frequently expected disruptive weather patterns caused by climate change. The current research presented the need for a multifaceted approach in minimizing mycotoxin exposure, most especially FM exposure, other than use of regulatory limits. Future studies should address the occurrence of other potentially harmful mycotoxins in these commodities.

## Figures and Tables

**Table 1:** Frequencies of *Fusarium*, *Aspergillus*, and *Penicillium* associated with maize, finger millet, pearl millet, and sorghum grain samples collected across Zimbabwe during two seasons.

Crop	Fungal Incidence (%) ^a^	Fungal Genera Frequency (%)			
		*Fusarium*	*Aspergillus*	*Penicillium*	Others
Pearl millet	40.5	4.3	3.5	0.6	91.6
Sorghum	40.2	17.3	4.4	1.3	77.1
Finger millet	54.8	6.8	4.2	1.2	73.5
Maize	72.6	57.5	14.3	11.7	16.5
Average	52.0	21.5	6.6	3.7	64.7

^a^ Fungal incidence was computed as number of kernels with visible growth of the corresponding genera divided by total number of plated kernels × 100.

**Table 2:** Frequency of *Aspergillus* species isolated from maize, finger millet, pearl millet, and sorghum grain samples collected in Zimbabwe during two seasons.

Crop	N ^a^	*Aspergillus* Species Frequency (%) ^c^				
		*A. flavus* ^b^	S morphotype	*A. parasiticus*	*A. ochraceous*	*A. niger*
Finger millet	122	75.4	12.3	11.5	0.0	1.1
Pearl millet	78	75.6	15.4	7.7	0.0	1.3
Sorghum	83	75.9	9.6	13.3	0.0	1.2
Maize	457	67.4	10.3	15.8	5.9	0.7

^a^ Total number of fungal isolates recovered per crop. ^b^ *Aspergillus flavus* L. morphotype. ^c^ Species frequency was calculated for each crop as the total number of each *Aspergillus* spp. isolated divided by the total number of fungal isolates × 100.

**Table 3:** Aflatoxin and fumonisin prevalence in maize, sorghum, pearl millet, and finger millet grain samples obtained from Zimbabwe during two cropping seasons along with the proportion of samples exceeding regional and international regulatory limits for human consumption.

Toxin	Crop ^a^	%P ^b^	Toxin Range (µg/kg)	Proportion of Samples Exceeding Limits (%)		
				EU ^c^	Zimbabwe ^d^	US-FDA ^e^
Aflatoxin	Maize (800)	51.2	ND ^f^–1369	19.0	11.0	8.0
	Finger millet (60)	13.3	ND–3.6	0.0	0.0	0.0
	Sorghum (60)	25.0	ND–4.3	3.3	3.3	2.0
	Pearl millet (60)	21.7	ND–58.0	1.7	0.0	0.0
Fumonisin	Maize (800)	88.9	ND–40,000	53.9	- ^g^	16.8
	Finger millet (60)	3.3	ND–1500	1.9	-	0.0
	Pearl millet (60)	0.0	ND	0.0	-	0.0
	Sorghum (60)	31.7	ND–2800	10.0	-	0.0

^a^ Number of samples in parentheses. ^b^ Proportion of positive samples with toxin concentration above limit of detection. ^c^ Samples with toxin concentration exceeding regulatory limit of the European Union (4 µg/kg for aflatoxin (AF) and 1000 µg/kg for fumonisin (FM)). ^d^ Samples with toxin concentration exceeding regulatory limit of Zimbabwe (10 µg/kg for AF). ^e^ Samples with toxin concentration exceeding regulatory limit of the United States Food and Drug Administration (20 µg/kg for AF and 4000 µg/kg for FM). ^f^ Not detected; limit of detection is 2 μg/kg (AF) and 300 μg/kg (FM). ^g^ Zimbabwe regulatory limit for FM is not available, therefore it was not computed.

**Table 4:** Levels of aflatoxin observed in maize samples collected at harvest and storage from different provinces of Zimbabwe during two seasons.

Province	District	N ^a^	Mean (µg/kg)	Weighted Means (µg/kg)	Range (µg/kg)	Proportion of Samples (%)			
						% *P* ^b^	>4 µg/kg ^c^	>10 µg/kg ^d^	>20 µg/kg ^e^
Manicaland	Buhera	70	15 ± 7	14.9 ± 10	ND ^f^–471	71.4	31.4	22.9	12.9
	Makoni	49	5 ± 1	6.1 ± 12	ND–54	73.5	22.4	10.2	6.1
	Mutare	93	7 ± 2	9.1 ± 9	ND–153	49.5	17.2	9.7	5.4
	Mutasa	74	6 ± 3	6.6 ± 10	ND–210	66.2	10.8	5.4	2.7
	Nyanga	77	3 ± 1	5.0 ± 10	ND–59	57.1	7.8	2.6	2.6
Mashonaland Central	Mt Darwin	97	7 ± 2	8.5 ± 8	ND–128	63.9	14.4	6.2	5.2
	Guruve	77	2 ± 0	4.8 ± 10	ND–32	63.6	2.6	1.3	1.3
	Muzarabani	97	87 ± 3	75.8 ± 9	ND–1369	83.5	44.3	33.0	27.8
	Rushinga	57	7 ± 2	8.8 ± 11	ND–55	71.9	24.6	15.8	12.3
Midlands	Chirumanzi	35	23 ± 2	20.1 ± 13	ND–693	68.6	20.0	8.6	5.7
	Gokwe South	47	5 ± 2	7.8 ± 12	ND–81	68.1	12.8	6.4	4.3
	Kwekwe	27	4 ± 0	9.9 ± 14	ND–67	70.4	7.4	3.7	3.7

^a^ Number of samples collected per district. ^b^ Proportion (%) of positive samples with aflatoxin concentration above limit of detection. ^c^ Samples with aflatoxin concentration exceeding regulatory limit of the European Union. ^d^ Samples with aflatoxin concentration exceeding regulatory limit of Zimbabwe. ^e^ Samples with aflatoxin concentration exceeding regulatory limit of the United States Food and Drug Administration. ^f^ Not detected; limit of detection is 2 μg/kg.

**Table 5:** Concentration of fumonisin (µg/kg) in maize samples collected over two cropping seasons (2015/2016 and 2016/2017) in three provinces of Zimbabwe.

Province	District	N ^a^	Means (µg/kg)	Weighted Means (µg/kg)	Range (µg/kg)	Proportion of Samples (%)		
						% P ^b^	>1000 (µg/kg) ^c^	>4000 (µg/kg) ^d^
Manicaland	Buhera	70	2200 ± 387	2337 ± 408	ND ^e^–9000	91.4	55.7	17.1
	Makoni	49	1067 ± 160	1537 ± 459	ND–5000	81.6	34.7	4.1
	Mutare	93	2027 ± 387	1991 ± 378	ND–28,000	86.0	46.2	10.8
	Mutasa	74	1326 ± 146	1785 ± 407	ND–6000	86.5	44.6	4.1
	Nyanga	77	1879 ± 257	1977 ± 395	ND–13,000	96.1	57.7	30.9
Mashonaland Central	Mt Darwin	97	3501 ± 439	3107 ± 330	ND–19,000	91.8	69.1	27.8
	Guruve	77	2810 ± 396	2455 ± 388	ND–18,000	92.2	66.2	20.8
	Muzarabani	97	3472 ± 508	3250 ± 384	ND–34,000	91.8	57.7	30.9
	Rushinga	57	3254 ± 778	3180 ± 453	ND–40,000	87.7	61.4	24.6
Midlands	Chirumanzi	35	1680 ± 413	1757 ± 511	ND–11,500	68.6	45.7	8.6
	Gokwe South	47	2249 ± 736	2286 ± 463	ND–33,000	83.0	42.6	12.8
	Kwekwe	27	967 ± 236	1119 ± 548	ND–5100	70.4	33.3	3.7

^a^ Number of samples in the category. ^b^ Proportion (%) of samples with fumonisin concentration above limit of detection (LOD). ^c^ Samples with fumonisin concentration exceeding regulatory limit of the European Union. ^d^ Samples with fumonisin concentration exceeding regulatory limit of the United States Food and Drug Administration (US-FDA). ^e^ ND = not detected; LOD is 300 μg/kg.

**Table 6:** Average concentration of aflatoxin, and dietary exposure for total aflatoxin and aflatoxin B1 in maize, sorghum, pearl millet, and finger millet grain samples that were produced and traded by farming households in different provinces of Zimbabwe during two seasons (2015/2016 and 2016/2017).

Aflatoxin Category	Crop	Mean (µg/kg) ^a^	Dietary Exposure (ng/kg bw/d)			MOE ^c^	Population Risk ^d^
			PDI (Range)	PDI (Mean)	MPDI (P95) ^b^		
Total aflatoxin	Maize	17.2	8.2–5593	70.141	206.7	2.42	-
	Finger millet	2.1	0.3–0.5	0.298	0.4	570.47	-
	Pearl millet	3.2	0.3–8.3	0.461	0.47	368.76	-
	Sorghum	2.3	0.6–1.3	0.670	1.1	253.73	-
Aflatoxin B1	Maize	8.6	4.1–2797	35.071	103.4	4.85	1.815
	Finger millet	1.0	0.1–0.3	0.149	0.2	1140.94	0.008
	Pearl millet	1.6	0.1–4.2	0.230	0.24	739.13	0.012
	Sorghum	1.1	0.3–0.7	0.335	0.5	507.46	0.017

^a^ Unweighted mean values for total aflatoxins (AF) and the estimated aflatoxin B1 (AFB1) obtained from pooled aflatoxin data sets for both seasons due to the nonsignificant differences observed across seasons and sampling time. AFB1 was estimated by halving AF detected in each sample. ^b^ 95th percentile for each category. ^c^ Margin of exposure. ^d^ Number of persons within the population at risk of developing liver cancer per year (cancer/year/100,000 population); risk not computed for AF but limited to AFB1.

**Table 7:** Dietary exposure to total aflatoxin and the estimated aflatoxin B1 attributed to consumption of maize that were produced and traded in different parts of Zimbabwe during two cropping seasons (2015/2016 and 2016/2017).

Aflatoxin Category	AEZ ^a^	%P ^b^	Mean (µg/kg) ^c^	Dietary Exposure (ng/kg bw/d)			MOE ^g^	Population Risk ^h^
				PDI (Range) ^d^	PDI ^e^	MPDI (P95) ^f^		
Total aflatoxin	IIA	26.9	2.6	8–82	10.708	19.203	15.876	-
	IIB	53.9	5.2	8–503	21.236	33.502	8.005	-
	III	43.3	6.8	8–2831	27.612	49.845	6.157	-
	IV	66.1	38.5	8–5593	157.475	506.214	1.080	-
Aflatoxin B1	IIA	-	1.3	4–41	5.354	9.601	31.752	0.277
	IIB	-	2.6	4–251	10.618	16.751	16.011	0.550
	III	-	3.4	4–1416	13.806	24.923	12.313	0.715
	IV	-	19.3	4–2797	78.738	253.107	2.159	4.075

^a^ Represents agroecological zones with precipitation levels of 450–650 mm (IV), 650–800 mm (III), and 750–1000 mm (IIA and IIB). ^b^ Proportion (%) of samples with aflatoxin B1 (AFB1) concentration above limit of detection. ^c^ Unweighted mean values for total aflatoxins and the estimated AFB1 obtained from pooled aflatoxin data for both seasons due to the nonsignificant differences observed across seasons and sampling time. AFB1 was estimated by halving total aflatoxins detected in each sample. ^d^ Probable daily intake. ^e^ Average probable daily intake. ^f^ Maximum probable daily intake; 95th percentile for each category. ^g^ Margin of exposure. ^h^ Number of persons within the population at risk of developing liver cancer per year (cancer/year/100,000 population); often exposure risks are computed using AFB1 data, so it was not computed for total aflatoxin.

**Table 8:** Dietary exposure to total aflatoxin and the estimated aflatoxin B1 attributed to consumption of maize produced and traded across different districts of Zimbabwe during the 2015/2016 and 2016/2017 cropping seasons.

Category	District	Mean (µg/kg) ^a^	Dietary Exposure (ng/kg bw/d)			MOE ^e^	Population Risk ^f^
			PDI (Range) ^b^	PDI ^c^	MPDI (P95) ^d^		
Total aflatoxin	Buhera	15.6	8–1924	63.596	261.074	2.673	-
	Chirumanzi	23.8	8–2831	97.040	81.713	1.752	-
	Darwin	6.9	8–524	28.393	49.845	5.987	-
	Gokwe South	5.0	8–329	20.350	77.628	8.354	-
	Guruve	2.7	8–129	11.015	13.891	15.433	-
	Kwekwe	4.8	8–273	19.763	27.783	8.602	-
	Makoni	5.4	8–219	22.113	83.348	7.688	-
	Mutare	7.4	8–625	30.102	81.713	5.647	-
	Mutasa	6.6	8–858	27.087	33.911	6.276	-
	Muzarabani	87.0	8–5593	355.327	2320.659	0.478	-
	Nyanga	3.7	8–243	15.006	20.428	11.329	-
	Rushinga	7.1	8–226	29.173	136.870	5.827	-
Aflatoxin B1	Buhera	7.8	4–962	31.798	130.537	5.346	1.646
	Chirumanzi	11.9	4–1416	48.520	40.857	3.504	2.511
	Darwin	3.5	4–262	14.197	24.923	11.974	0.735
	Gokwe South	2.5	4–164	10.175	38.814	16.708	0.527
	Guruve	1.3	4–64	5.508	6.946	30.864	0.570
	Kwekwe	2.4	4–137	9.881	13.891	17.205	1.023
	Makoni	2.7	4–110	11.056	41.674	15.376	1.145
	Mutare	3.7	4–313	15.051	40.857	11.295	1.558
	Mutasa	3.3	4–429	13.543	16.956	12.553	0.701
	Muzarabani	43.5	4–2797	177.663	1160.329	0.957	9.196
	Nyanga	1.8	4–121	7.503	10.214	22.658	0.388
	Rushinga	3.6	4–113	14.587	68.435	11.654	0.755

^a^ Unweighted mean values for total aflatoxin (AF) and the estimated aflatoxin B1 (AFB1) obtained from pooled aflatoxin data for both seasons due to the nonsignificant differences observed across seasons and sampling time. AFB1 was estimated by halving AF detected in each sample. ^b^ Probable daily intake. ^c^ Average probable daily intake. ^d^ Maximum probable daily intake; 95th percentile for each category. ^e^ Margin of exposure. ^f^ Number of persons within the population at risk of developing liver cancer per year (cases/100,000 population/year); often exposure risks are computed using AFB1 data, so it was not computed for total AF.

**Table 9:** Estimated potential health and trade impact of aflatoxin B1 contamination of stored maize grains in different provinces of Zimbabwe following the 2015/2016 and 2016/2017 cropping seasons.

Regulatory Limit ^a^	Province	Mean Concentration (µg/kg)	% Rejections (For Human Consumption)	Dietary Exposure/PDI (ng/kg bw/d)	Population Risk ^b^
No Limit	Manicaland	3.6	0.0	14.8	0.8
	Mashonaland	10.1	0.0	41.1	2.1
	Midlands	1.7	0.0	7.0	0.4
ALARA	Manicaland	1.4	5.1	5.7	0.3
	Mashonaland	1.9	5.3	7.7	0.4
	Midlands	1.2	5.6	4.8	0.3
Provisional	Manicaland	1.4	5.1	5.7	0.3
	Mashonaland	1.5	8.6	6.0	0.3
	Midlands	1.5	1.9	6.2	0.3
EU	Manicaland	1.1	10.3	4.5	0.2
	Mashonaland	1.2	13.9	4.7	0.2
	Midlands	1.1	7.4	4.6	0.2

^a^ EU maximum limit for unprocessed maize grain is 4 µg/kg; while provisional is 10 µg/kg for Zimbabwe. ^b^ Represents the number of persons within the population at risk of developing liver cancer per year (cancer cases/100,000 persons/year), estimated using Equation (3).

## Data Availability

The data presented in this study are available on request from the corresponding author. The data are not publicly available due to privacy issues.
